# Establishment of an assistive diagnostic model for schizophrenia with oxidative stress biomarkers

**DOI:** 10.3389/fphar.2023.1158254

**Published:** 2023-03-15

**Authors:** Shuying Wang, Xiuxia Yuan, Lijuan Pang, Peilun Song, Rufei Jia, Xueqin Song

**Affiliations:** ^1^ Department of Psychiatry, First Affiliated Hospital of Zhengzhou University, Zhengzhou, China; ^2^ Biological Psychiatry International Joint Laboratory of Henan/Zhengzhou University, Zhengzhou, China; ^3^ Henan Psychiatric Transformation Research Key Laboratory/Zhengzhou University, Zhengzhou, China; ^4^ School of Information Engineering, Zhengzhou University, Zhengzhou, China

**Keywords:** schizophrenia, oxidative stress, SOD, indirect bilirubin, objective diagnostic model

## Abstract

**Objective:** In this study, alterations in oxidative stress-related indicators were evaluated in drug-naïve, first-episode schizophrenia (SCZ) patients, and the effectiveness of blood serum glucose, superoxide dismutase (SOD), bilirubin in the objective assistive diagnosis of schizophrenia was explored.

**Materials and methods:** We recruited 148 drug-naïve, first-episode SCZ patients and 97 healthy controls (HCs). Blood biochemical indexes including blood glucose, SOD, bilirubin and homocysteine (HCY) in participants were measured, the indexes were compared between patients with SCZ and HCs. The assistive diagnostic model for SCZ was established on the basis of the differential indexes.

**Results:** In SCZ patients, the blood serum levels of glucose, total (TBIL), indirect bilirubin (IBIL) and homocysteine (HCY) were significantly higher than those in HCs (*p* < 0.05), and the serum levels of SOD were significantly lower than those in HCs (*p* < 0.05). There was a negative correlation between SOD with the general symptom scores and total scores of PANSS. After risperidone treatment, the levels of uric acid (UA) and SOD tended to increase in patients with SCZ (*p* = 0.02, 0.19), and the serum levels of TBIL and HCY tended to decrease in patients with SCZ (*p* = 0.78, 0.16). The diagnostic model based on blood glucose, IBIL and SOD was internally cross-validated, and the accuracy was 77%, with an area under the curve (AUC) of 0.83.

**Conclusion:** Our study demonstrated an oxidative state imbalance in drug-naïve, first-episode SCZ patients, which might be associated with the pathogenesis of the disease. Our study proved that glucose, IBIL and SOD may be potential biological markers of schizophrenia, and the model based on these markers can assist the early objective and accurate diagnosis of schizophrenia.

## 1 Introduction

Schizophrenia (SCZ) is a group of chronic severe brain disorders with extremely high disability rates, with a lifetime prevalence of up to 1% (Owen et al., 2016) and 12, 767, 900 years of healthy life lost due to disability (YLDs) (Spencer et al., 2018). Patients can exhibit positive symptoms, negative symptoms, and cognitive, emotional, behavioural, and other impairments in many aspects of life and even irreversible brain damage, resulting in reduced quality of life and reduced social functioning, thus placing a heavy burden on families and society. Early treatment can lead to better outcomes, but early diagnosis and timely treatment are needed. However, certain standard criteria need to be met regarding symptoms, such as the presence of psychotic symptoms, a combination of “positive” symptoms and “negative” symptoms (Smeland et al., 2020) and cognitive impairment, and the course of the illness for a diagnosis of SCZ to be made.

However, the symptoms of SCZ are typical only in the symptomatic stage, and the symptoms of SCZ in the prodromal stage are often not obvious, as in this stage, patients may show only changes in mood, cognition, behaviour, physical symptoms, etc., or even only compulsive behaviour, which leads to difficulties in the early diagnosis and treatment of SCZ. The best time to treat mental illness is before symptoms appear, and for early intervention, biomarkers that can guide medical staff to identify patients earlier and to achieve secondary prevention are needed. To achieve these goals, it is necessary to find a group of biomarkers to establish objective and early diagnostic models.

In the field of mental disorders, biomarkers mainly include neuroimaging and central and peripheral biochemical indicators. Imaging techniques have been applied to explore abnormalities in white matter connections, grey matter, and cognitive and emotional neural circuits. These abnormalities may appear in SCZ patients (Fannon et al., 2000). Central biochemical indicators are mainly found in cerebrospinal fluid (CSF), while peripheral biochemical indicators can be found peripheral blood, saliva, urine, faeces, etc., but blood often contains more information. CSF seems to be a better source of biomarkers of mental disorders than blood. However, due to ethical and other restrictions, it is unrealistic to test the CSF of patients. However, there are many connections between peripheral blood and CSF biochemical indexes. For example, the expression levels of a large number of miRNAs in CSF and peripheral blood (Martinez and Peplow, 2020), the state of oxidative metabolism indicators is consistent in CSF and peripheral blood, an increase in oxidized protein occurs in both cerebrospinal fluid and peripheral blood in patients with amyotrophic lateral sclerosis (Siciliano et al., 2007), and peripheral blood has its own advantages; for instance, peripheral blood is accessible, quantifiable, and economical (Wu et al., 2022). Inflammatory markers such as interleukin-6 (IL-6), interferon-γ (IFN-γ), and tumour necrosis factor-α (TNF-α) were found to be significantly higher in first-episode psychosis patients than in controls (Flatow et al., 2013; Ding et al., 2014; Fraguas et al., 2019), whereas brain-derived neurotrophic factor (BDNF) levels were found to be lower in first-episode SCZ patients (ShoshinaHovis et al., 2021). However, many indicators are not universal or common, and their measurement is expensive. Therefore, when establishing auxiliary diagnostic models, the general inclination is to use clinically simple, available, economical, and universal indicators as diagnostic indexes that can be applied in both clinical decision-making and auxiliary diagnosis.

For example, uric acid (UA), bilirubin, albumin (ALB), folate, superoxide dismutase (SOD) and blood glucose are common biochemical indicators in the clinic. Previous studies have shown that the serum UA and total bilirubin (TBIL) levels in SCZ patients at the acute stage are higher than those in healthy controls (HCs) (Müller et al., 1991; Solberg et al., 2019; Lu et al., 2020), while the serum ALB level is lower in SCZ patients (Yao et al., 2000; Solberg et al., 2019). However, some studies have shown that bilirubin is reduced in SCZ patients (Yao et al., 2000; Pae et al., 2004). Relatively uniformly, articles often explain these results from the perspective of oxidative stress: because UA is a selective antioxidant, removing superoxide by preventing the degradation of SOD can prevent oxidative stress from spreading from the extracellular environment to the intracellular environment by maintaining the integrity of the plasma membrane at the lipid-water interface (Guerreiro et al., 2009; Yao and Keshavan, 2011). Therefore, a decrease in plasma UA may reflect a decrease in the ability to prevent superoxide and peroxynitrite from damaging cellular components. However, it remains unclear whether UA levels contribute to the cause or are simply a consequence of these pathological conditions (Kutzing and Firestein, 2008). An increase in serum bilirubin may be the result of an increase in erythrocyte membrane fragility under oxidative stress, which leads to the conversion of haemoglobin into bilirubin (Glen et al., 1994; Radhakrishnan et al., 2011). In addition, the role of bilirubin in the central nervous system is complex. Moderate and high concentrations of bilirubin can induce neuronal and glial cell damage, while low concentrations of bilirubin can play an antioxidant protection role (Doré et al., 1999; Pommerening Dornelles et al., 2019).

Oxidative stress refers to an imbalance in pro-oxidants and antioxidants and the related redox circuit destruction and macromolecular damage (Patel, 2016), which is a pathological state. Oxidative stress is often considered to be related to SCZ (Flatow et al., 2013). 90% of the brain’s energy demand is provided by aerobic process, which is rich in easily oxidized unsaturated lipids (Lohr and Browning, 1995; Ermakov et al., 2021). The brain is highly sensitive to oxidative stress and increased reactive oxygen species produced during neuroinflammatory processes (Naureen et al., 2022). However, excessive oxidation damages the cell membrane and leads to mitochondrial dysfunction and metabolic abnormality, which eventually leads to abnormal nerve development, abnormal myelination and neurotransmitter transmission, which are related to the pathogenesis of schizophrenia (Ermakov et al., 2021). Oxidative stress in hypothalamus interferes with the function of hypothalamus-pituitary-adrenal axis (HPA-axis) and promotes the development of mental diseases (Vetulani, 2013; Valli et al., 2016). Some recent genetic polymorphisms of oxidative stress related to schizophrenia further illustrate the potential relationship between oxidative stress and schizophrenia, but it is still unknown whether oxidative stress is the cause or result of schizophrenia (Chowdari et al., 2011).

To verify the role of these biochemical indicators, which are associated with oxidative stress and are economical and common, in the diagnosis of SCZ, we recruited drug-naive, first-episode SCZ patients and tested their blood sugar, intact bilirubin (IBIL), SOD, UA and other blood indexes. Finally, we established a diagnostic model for early SCZ that included different blood indexes and evaluated the role of these blood indexes in the onset of SCZ.

## 2 Methods

### 2.1 Participants

The research participants were drug-naïve, first-episode SCZ patients in the psychiatric inpatient department of the First Affiliated Hospital of Zhengzhou University from 2015 to 2019. During the same time period, HC participants were recruited from local communities through advertisements. This study was approved by the Ethics Committee of the First Affiliated Hospital of Zhengzhou University. All participants were recruited between the ages of 18 and 45. Each participant signed a written informed consent form. Patient inclusion criteria were as follows: (1) a diagnosis of first-episode SCZ based on the Diagnostic and Statistical Manual of Mental Disorders fifth edition (DSM-5) criteria, which was confirmed by an attending psychiatrist and a researcher (a graduate student or a doctoral student) and further confirmed through a structured clinical interview administered by a research psychiatrist (X.S, a professor, chief psychiatrist) using the DSM-5; (2) no prescription medication use at the time of enrolment; and (3) a total Positive and Negative Syndrome Scale (PANSS) score ≥60 points (Keepers et al., 2020). Patient exclusion criteria were as follows: (1) a diagnosis of an autoimmune disease, heart disease, hepatobiliary or gastrointestinal disease, haematological disease, diabetic nervous system disease or mental disease other than first-episode SCZ; and (2) pregnancy or lactation. The exclusion criteria for HCs were the same as those for patients; in addition, HCs did not have any history of mental illness.

Patients were treated with risperidone, and the dosage was gradually titrated from 1 mg/day to 4–6 mg/day as clinically indicated. After 24 weeks of treatment, blood samples were collected, and PANSS scores were evaluated in the outpatient department.

### 2.2 Research process

At baseline, a comprehensive clinical evaluation was conducted, including a detailed medical history and mental history, a physical examination and the PANSS (for patients). Fasting blood samples were collected from patients and HCs at baseline, and fasting blood samples were collected again, along with PANSS scores, at the 24th week after treatment.

### 2.3 Measurement of metabolic parameters

Serum levels of glucose, total cholesterol (TCHO), triglyceride (TG), high-density lipoprotein (HDL), and low-density lipoprotein (LDL), UA, TBIL, IBIL and direct bilirubin (DBIL)were analyzed using standard enzymatic methods and an automated analyzer (Roche Diagnostics, C8000, Germany). Serum levels of homocysteine (HCY) were detected using an immune turbidimetric test (Roche Cobas c501, Switzerland). Serum levels of SOD (MEIMIAN, MM-0135H) were measured by a chemical colorimetry assay using a Roche automatic biochemical analyzer (Roche Diagnostics, C8000, Germany). All assays were performed according to the manufacturer’s instructions.

### 2.4 Statistical analysis

IBM SPSS Statistics for Windows (version 26) was used to analyse the data. The chi-square test was used to compare categorical variables. The Shapiro–Wilk normality test was used to compare continuous variables. Not all demographic data nor biochemical index data showed a Gaussian distribution; thus, comparisons between patients with SCZ and HCs were performed *via* independent-sample *t* tests (data with a normal distribution) and the Mann–Whitney *U* test (data with a skewed distribution), Pearson or Spearman correlation analysis was used between PANSS scores and biochemical indexes. Comparisons between 24 weeks of treatment and HCs were the same as above. Paired sample *t*-test was used to detect the differences of indexes before and after treatment in SCZ patients. A *p*-value < 0.05 was considered significant. We used Enter Selection of binary logistic regression models to distinguish SCZ patients and HCs with selected biochemical indexes, with criterion of *p*-value 0.05 for entry. The Scikit-learn (sklearn) tool of Python (computer programming language) and logistic regression model were used to establish the diagnostic model for SCZ and to train the machine to distinguish between patients with SCZ and HCs on the basis of the different blood indexes related to oxidative stress. The total sample was randomly divided into a training set and a testing set at a ratio of 9:1. The training set was used to train the machine learning model, and the testing set was used for internal cross validation of the model. The model was run 100 times to obtain the average diagnostic model, accuracy, and receiver-operating characteristic (ROC) curve.

## 3 Results

### 3.1 Comparison of patients with SCZ and HCs

A total of 245 individuals participated (including 148 drug-naïve, first-episode SCZ patients and 97 HCs) in this study. Blood serum levels of glucose (Z = 3.983, *p* = 6.80*10^–5^), TBIL (Z = 2.950, *p* = 3*10^–3^), IBIL (Z = 4.416, *p* = 1.00*10^–5^) and HCY (Z = 4.311, *p* = 1.60*10^–5^) were significantly higher in SCZ patients than in HCs. The levels of SOD (Z = −6.167, *p* = 6.95*10^–10^) in SCZ patients were significantly lower than in HCs. We did not find significant differences related to age, sex, education level, smoking status, or body mass index (BMI) between SCZ patients and HCs (Table 1).

**TABLE 1:** Demographic characteristics and peripheral biochemical indicators of patients with SCZ and HCs.

Variable		Patients with SCZ (*n* = 148)	HCs (*n* = 97)	Z/t/X^2^	*p*
Sex	Male	59	37	0.073	0.787
	Female	89	60		
Smoking status	Yes	4	4	0.375	0.541
	No	144	93		
Age		24.2 ± 6.62	23.1 ± 1.81	−1.337	0.181
Weight		58.46 ± 10.44	59.05 ± 11.06	−0.229	0.819
BMI*		20.86 ± 2.81	21.12 ± 2.61	−0.853	0.393
Glucose		4.49 ± 0.47	4.22 ± 0.83	3.983	6.80*10^–5^
UA		271.55 ± 74.64	274.99 ± 70.02	−0.454	0.65
TBIL		10.40 ± 4.97	8.60 ± 4.07	2.950	3*10^–3^
DBIL		4.60 ± 2.05	4.40 ± 1.80	0.745	0.456
IBIL		5.83 ± 3.18	4.21 ± 2.48	4.416	1.00*10^–5^
TCHO		3.67 ± 0.73	3.73 ± 0.68	−0.561	0.575
TG		0.87 ± 0.41	0.97 ± 0.50	−1.867	0.062
HDL		1.32 ± 0.30	1.31 ± 0.36	0.681	0.496
LDL		2.12 ± 0.60	2.04 ± 0.50	0.724	0.469
SOD		187.45 ± 22.56	210.89 ± 31.50	−6.167	6.95*10^–10^
HCY		18.54 ± 13.81	15.56 ± 11.77	4.311	1.60*10^–5^

*BMI: Body Mass Index; UA: Uric acid; TBIL:Total Bilirubin; DBIL: Direct Bilirubin; IBIL: Indirect Bilirubin; TCHO: Total Cholesterol; TG: Triglyceride; HDL: High-density Lipoprotein; LDL: Low-density lipoprotein; SOD: Superoxide Dismutase; HCY: Homocysteine.

### 3.2 Correlation analyses within the SCZ patients

At baseline, there was a negative correlation between SOD with the general symptom scores and total scores of PANSS (r = −0.183, *p* < 0.05; r = −0.189, *p* < 0.05). After 24 weeks of treatment, there was a negative correlation between IBIL at baseline and the change of the negative symptom scores (r = −0.338, *p* < 0.05), and a negative correlation between HCY at baseline and the change of positive symptom scores (r = - 0.360, *p* < 0.05).

### 3.3 Comparison of indexes at baseline and after treatment

After 24 weeks of treatment, several biochemical indexes changed in SCZ patients. BMI (Z = −5.95, *p* = 1.6*10^–8^) and serum levels of UA (Z = −4.88, *p* = 2*10^–5^) increased after 24 weeks of risperidone treatment, serum levels of DBIL (Z = 4.05, *p* = 2.4*10^–4^) decreased after treatment. Blood glucose (Z = −1.16, *p* = 0.25), SOD (Z = −1.28, *p* = 0.21), and IBIL (Z = −0.62, *p* = 0.54) in SCZ patients showed a slight trend to increase and TBIL (Z = 1.76, *p* = 0.78) and HCY (Z = 1.39, *p* = 0.16) showed a slight trend to decrease after 24 weeks of risperidone treatment, but the difference was not significant. After 24 weeks of treatment, the clinical symptoms assessed by the PANSS (*p*: Z = 7.3, *p* = 2.8*10^–13^; N: Z = 5.41, *p* = 6.3*10^–8^; G: Z = 6.242, *p* = 4.3*10^–10^; T: Z = 7.00, *p* = 2.7*10^–12^) were significantly decreased compared with their levels at baseline (Table 2).

**TABLE 2:** Peripheral biochemical indicators at baseline and after treatment.

Variable	Baseline (*n* = 39)	After treatment (*n* = 39)	t/Z	*p*
BMI	20.64 ± 2.00	23.21 ± 2.97	−5.95	1.6*10^–8^
Glucose	4.47 ± 0.47	4.58 ± 0.46	−1.16	0.25
UA	263.49 ± 74.56	305.67 ± 83.46	−4.88	2*10^–5^
TBIL	10.15 ± 4.69	8.44 ± 2.42	1.76	0.78
DBIL	4.39 ± 1.59	3.27 ± 1.32	4.05	2.4*10^–4^
IBIL	5.76 ± 3.25	6.58 ± 9.26	0.62	0.54
SOD	188.99 ± 22.84	195.05 ± 20.96	−1.28	0.21
HCY	19.91 ± 15.85	14.09 ± 5.57	1.39	0.16
P*	20.64 ± 4.04	9.08 ± 2.82	7.3	2.8*10^–13^
N	21.03 ± 4.80	12.95 ± 5.33	5.41	6.3*10^–8^
G	40.64 ± 7.53	26.11 ± 7.15	6.242	4.3*10^–10^
T	82.31 ± 10.90	48.14 ± 12.87	7.00	2.7*10^–12^

*P: Positive symptom scores of PANSS; N: Negative symptom scores of PANSS; G: General pathological symptom scores of PANSS; T: Total scores of PANSS.

### 3.4 Comparison of indexes at SCZ after treatment and HCs

After 24 weeks of treatment, Body Mass Index (BMI) (Z = 3.77, *p* = 1.6*10^–4^) of SCZ patients was significantly higher than HCs. Blood serum levels of glucose (Z = 3.43, *p* = 6.1*10^–4^), UA (Z = 2.18, *p =* 0.03), IBIL (Z = 2.49, *p =* 1.3*10^–2^) were significantly higher in SCZ patients after treatment than in HCs. The levels of SOD (Z = −3.06, *p* = 2.2*10^–3^) and DBIL (Z = −3.39, *p* = 6.9*10^–4^) were significantly lower in SCZ patients after treatment than in HCs. The levels of TBIL (Z = −0.03, *p* = 0.98) and HCY (Z = −1.79, *p* = 0.07) in SCZ patients after treatment seemed higher than in HCs, but the results were not significant (Table 3).

**TABLE 3:** Peripheral biochemical indicators at SCZ after treatment and HCs.

Variable	After treatment (*n* = 39)	HC(*n* = 97)	t/Z	*p*
BMI	23.21 ± 2.97	21.12 ± 2.61	3.77	1.6*10–4
glucose	4.58 ± 0.46	4.22 ± 0.83	3.43	6.1*10–4
UA	305.67 ± 83.46	274.98 ± 70.02	2.18	0.03
TBIL	8.44 ± 2.42	8.60 ± 4.07	−0.03	0.98
DBIL	3.27 ± 1.32	4.40 ± 1.80	−3.39	6.9*10–4
IBIL	6.58 ± 9.26	4.21 ± 2.48	2.49	1.3*10–2
SOD	195.05 ± 20.96	210.89 ± 31.5	−3.06	2.2*10–3
HCY	14.09 ± 5.57	15.56 ± 11.77	−1.79	0.07

### 3.5 Regression analysis to identify patients with SCZ and HCs based on different indicators

#### 3.5.1 Based on glucose, IBIL, and SOD

**FIGURE 1:**
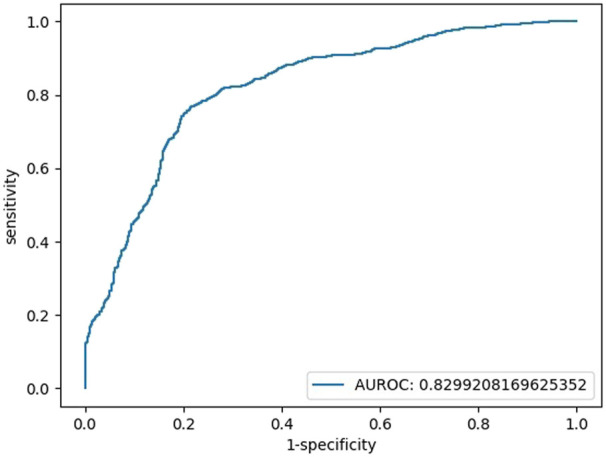
Testing set ROC curve of the early diagnosis of schizophrenia with the features of glucose, IBIL, and SOD.

When HCY was used as a feature, the coefficient was not significant (HCY’ *p* = 0.161, while the *p* values of glucose, IBIL, SOD, and constant were all below 0.05; HCY’ *p* = 0.113, while the *p* values of glucose, TBIL, and SOD, were constantly all below 0.05). Finally, we chose blood glucose, IBIL and SOD as our features. We simulated the logistic regression model of the sklearn tool with a 9:1 training set and test set by machine learning and repeated the run 100 times to obtain the average model. There was no multicollinearity between independent variables. The tolerance (TOL) of these factors was above 0.1, and their variance inflation factors (VIFs) were far below 3 (Supplementary Table S1). The binary regression model included 245 individuals (148 patients with SCZ vs. 97 HCs), with an area under the curve (AUC) of 0.83, an accuracy of 77%, a sensitivity of 79% and a specificity of 77% (Figure 1). The models are listed as follows:

Logit(*p*) = 3.70 + 0.97*[glucose]+0.25*[IBIL]-0.05 [SOD], Logit(*p*) = Ln [*p*/(1-P)], sensitivity = 0.77, specificity = 0.79, accuracy = 0.77, AUC = 0.83.

#### 3.5.2 Based on glucose, TBIL, and SOD

**FIGURE 2:**
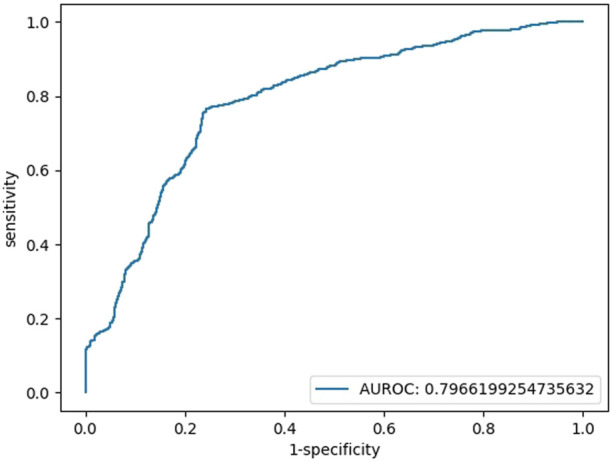
Testing set ROC curve of the early diagnosis of schizophrenia with the features of glucose, TBIL, and SOD.

We also repeated the above operations with the features of blood glucose, TBIL, and SOD, and there was no multicollinearity among independent variables (Supplementary Table S2). The AUC of the testing set in the final binary regression model was 0.80, with an accuracy of 76%, a sensitivity of 76% and a specificity of 76% (Figure 2). The models are listed as follows:

Logit(*p*) = 3.62 + 1.00*[glucose]+0.10*[TBIL]-0.05 [SOD], Logit(*p*) = Ln [*p*/(1-P)], sensitivity = 0.76, specificity = 0.76, accuracy = 0.76, AUC = 0.80.

## 4 Discussion

Evidence for a pathological connection between oxidative stress and SCZ has been shown. Changes in several oxidative stress-related indicators, such as UA, bilirubin, ALB, folic acid, SOD, and blood glucose, have also been confirmed to occur in SCZ patients (Yao and Keshavan, 2011; Patel, 2016; Lu et al., 2020; Huang et al., 2021). These indicators are not only oxidative stress-related indicators but also blood biochemical indicators commonly used in psychiatry. Our study identified blood biochemical indicators differentiating SCZ patients from HCs, and the differences can be explained from the perspective of oxidative stress. In addition, this study established an auxiliary diagnostic model for SCZ using these differential indicators, showing high credibility and potentially a role in early diagnosis and early treatment for some patients with early and difficult-to-identify SCZ (Wayner et al., 1987; Müller et al., 1991; Yao et al., 2000; Pae et al., 2004; Radhakrishnan et al., 2011; Patel, 2016; Solberg et al., 2019; Lu et al., 2020). At present, many researchers believe that the mechanism underlying SCZ development is related to nerve connections, intestinal microbes, genes and nerve development, and some biomarkers have been proposed. For example, reduced cortical grey matter thickness was found to be related to neuropathological findings (Cannon et al., 2015); abnormal functional connectivity and reduced numbers of dendritic spines were confirmed in SCZ (Lawrie et al., 2002; Glausier and Lewis, 2013; Konopaske et al., 2014; Cannon et al., 2015); and the haemolytic activities of the C1, C3, and C4 complement components in the serum of SCZ patients were significantly higher, with serum levels of C3 and C4 suggested as biological markers of acute negative symptoms of paranoid SCZ (Hakobyan et al., 2005; Morera et al., 2007). *Eggerthella* and *Lactobacillus* were found to be frequently higher, and *Coprococcus* frequently lower in SCZ patients than controls (McGuinness et al., 2022). Although recent studies have provided biological indicators for the diagnosis of SCZ in many aspects, it is still difficult to make an objective diagnosis of SCZ on the basis of these indicators as their measurement is expensive and difficult to implement. In this study, available common clinical information was used to distinguish SCZ patients from HCs, including haematological examination data, which are more universally obtained and have more clinical value. The results obtained were quite satisfactory. The AUC of this binary logistic regression model including blood glucose, IBIL and SOD was 0.83, and that of the logistic regression model including blood glucose, TBIL and SOD was 0.80. These features can provide good accuracy when used to distinguish patients with SCZ from HCs. Interestingly, the differences in IBIL and SOD showed a changing trend after treatment, but they were still significantly different from those of HCs; perhaps IBIL and SOD exist stably in the disease and therefore these indexes can serve as stable predictors.

In our research, SOD, an antioxidant, was significantly lower in SCZ patients than in HCs. SOD is the first line of defence against oxidation (Yao and Keshavan, 2011). The decrease in SOD means that the first line of defence against oxidation in drug-naïve, first-episode SCZ patients has been broken (Liu et al., 2022), and the body is about to be in a state of antioxidant decompensation, which needs urgent medical and physical treatment. In addition, in our research, the level of SOD at baseline was negatively correlated with the general pathological symptom scores and total scales in PANSS of the SCZ patients, which perhaps meant that stronger oxidative stress at baseline is related to more severe symptoms. Furthermore, SOD was elevated after 24 weeks of antipsychotic treatment, and symptoms improved, which means that the antioxidant capacity of patients improved after treatment (Yao and Keshavan, 2011; Rossetti et al., 2020). However, only 24 weeks of treatment cannot completely prevent the adverse effects of antioxidant imbalance due to the long-term oxidative stress of SCZ. In contrast, another antioxidant, UA, was not significantly different between SCZ patients and HCs, which might indicate that a non-enzymatic antioxidant system is still in place in drug-naïve, first-episode SCZ patients. After treatment, UA was significantly higher, even higher than in HCs, which perhaps meant the antioxidant capacity in patients with SCZ has been saved after treatment (Yao and Keshavan, 2011). The level of bilirubin in patients with SCZ is controversial. In our results, bilirubin was elevated in patients with drug-naïve, first-episode SCZ, which is consistent with some previous studies (Müller et al., 1991; Solberg et al., 2019; Lu et al., 2020). These findings show that there is excessive oxidative stress in drug-naïve, first-episode SCZ patients, but there is still an antioxidant defence mechanism, with compensatory elevation of antioxidant bilirubin in blood biochemistry (Radhakrishnan et al., 2011; Pommerening Dornelles et al., 2019). After treatment, DBIL tended to decrease, which means that 24 weeks of treatment improved the compensated elevation of bilirubin (Rigato et al., 2005).

At baseline, the levels of TBIL and IBIL in SCZ patients were higher than those in HCs, but there was no significant difference in DBIL. After treatment, DBIL in SCZ patients decreased significantly, even lower than HC. The level of IBIL tended to decrease, but it was not significant, and the level was still higher than in HCs. In other words, the downward trend of DBIL was greater than that of IBIL. First of all, the decrease of bilirubin might mean that risperidone treatment of SCZ could save the non-enzymatic antioxidant mechanism. Secondly, the decline rates of IBIL and DBIL are inconsistent, which may be due to the following reasons: IBIL is unconjugated bilirubin, mainly from broken red blood cells (Glen et al., 1994). However, red cells were in an oxygen-rich environment, mainly with a membrane structure, which was rich in unsaturated fatty acids, so red cells were prone to peroxidation. In SCZ patients with oxidative damage, there may be an increase in the destruction of red blood cells, so while IBIL is decreasing, it is also continuously produced, and it takes time for IBIL to be transformed into DBIL through the liver, which leads to the decrease of DBIL faster than IBIL. Of course, the sample size of follow-up is small, or it might be the bias caused by small samples. After treatment, the inconsistent levels of IBIL and DBIL in SCZ patients led to no significant difference in their total bilirubin levels in HCs. In addition, our research showed that the level of IBIL at baseline was negatively correlated with the change of negative symptom scores after 24 weeks of treatment, which might mean that the lower IBIL at baseline was related to better curative effect. Indeed, the level of IBIL may be cause or consequence of a psychotic state, it needs further study.

Similarly, the HCY at baseline was significantly higher than in HCs, but there was a downward trend after treatment, while it was not significant. However, the HCY at baseline was negatively correlated with the change of positive symptom scores of SCZ patients after treatment, which might mean that HCY was related to the onset of schizophrenia, and lower HCY meant better curative effect. Unfortunately, when HCY was substituted into the diagnosis model, its parameter was not significant, so HCY was not included in the model.

The changes of these indexes not only explained the changes of oxidative stress in SCZ, but also explained the improvement of risperidone on oxidative stress in SCZ patients. Studies had shown that risperidone may have antioxidant effects by reducing lipid peroxidation, improving SOD activity, reducing inflammation level, reducing brain glucose metabolism and reducing free radical expression and so on (Noto et al., 2015; Al-Amin et al., 2018; Casquero-Veiga et al., 2019; Mihai et al., 2021). However, risperidone also has the risk of obesity and endocrine disorders (Kaushal et al., 2012), so the changes of these oxidation indexes cannot be analysed from the perspective of oxidative stress alone, and the influence of lipid metabolism should still be considered. This also reminds us that the limitations of antipsychotic drugs and the complexity of their therapeutic mechanisms need more research to explore in the future.

Although these indicators show stability in the course of disease, there may still be some influencing factors. After 24 weeks of treatment, patients may show weight changes and drug-induced obesity, which may also affect patients’ blood glucose, IBIL and SOD because these features are related to metabolism and oxidative stress. Moreover, risperidone can affect the level of oxidative stress in patients. After treatment, the level of oxidative stress in patients has been shown to be affected by drugs, metabolism, inflammation and other factors (Liu et al., 2022). However, 24 weeks of treatment is insufficient for the long-term treatment of SCZ, and the impact may be weak. Furthermore, the progression of the disease itself will also affect lipid metabolism and oxidative stress. Therefore, more experiments are needed to verify the changes in these three features during SCZ. The three biochemical indexes used in this study are the most important features in the diagnostic model for SCZ and can help provide an objective clinical diagnosis. In the future, we will continue to expand the sample size to verify the accuracy and clinical significance of this model.

In order to make the model popular and easy to repeat, we chose the clinical common and widely used indicators to be included in the study. Nevertheless, the sample size is still small. If the research can be expanded and supported by more data, then this model will be economical and effective, especially for those patients with hidden symptoms of schizophrenia.

All in all, our study established an economical, practical, and innovative model to assist in the clinical diagnosis of SCZ. In this model, common clinically relevant indicators of oxidative stress were used to objectively diagnose SCZ, and these indexes are stable in the course of SCZ. These objective biochemical indicators were used to assist in the early diagnosis of SCZ and treat hidden psychotic symptoms in a timely manner to achieve a better prognosis and reduce the burden on the family and society. In addition, the model showed high credibility, could assist in the diagnosis of SCZ quite efficiently, and could play a role in early diagnosis and early treatment for some patients with early and difficult-to-identify SCZ.

## Data Availability

The raw data supporting the conclusions of this article will be made available by the authors, without undue reservation.

## References

[B1] Al-Amin M. M. Choudhury M. F. R. Chowdhury A. S. Chowdhury T. R. Jain P. Kazi M. (2018). Pretreatment with risperidone ameliorates systemic LPS-induced oxidative stress in the cortex and Hippocampus. Front. Neurosci. 12, 384. 10.3389/fnins.2018.00384 29937710 PMC6002684

[B2] Cannon T. D. Chung Y. He G. Sun D. Jacobson A. van Erp T. G. M. (2015). Progressive reduction in cortical thickness as psychosis develops: A multisite longitudinal neuroimaging study of youth at elevated clinical risk. Biol. psychiatry 77, 147–157. 10.1016/j.biopsych.2014.05.023 25034946 PMC4264996

[B3] Casquero-Veiga M. Garcia-Garcia D. MacDowell K. S. Perez-Caballero L. Torres-Sanchez S. Fraguas D. (2019). Risperidone administered during adolescence induced metabolic, anatomical and inflammatory/oxidative changes in adult brain: A pet and mri study in the maternal immune stimulation animal model. Eur. Neuropsychopharmacol. 29, 880–896. 10.1016/j.euroneuro.2019.05.002 31229322

[B4] Chowdari K. V. Bamne M. N. Nimgaonkar V. L. (2011). Genetic association studies of antioxidant pathway genes and schizophrenia. Antioxid. Redox Signal 15, 2037–2045. 10.1089/ars.2010.3508 20673164 PMC3159115

[B5] Ding M. Song X. Zhao J. Gao J. Li X. Yang G. (2014). Activation of Th17 cells in drug naïve, first episode schizophrenia. Prog. neuro-psychopharmacology Biol. psychiatry 51, 78–82. 10.1016/j.pnpbp.2014.01.001 24447943

[B7] Doré S. Takahashi M. Ferris C. D. ZakhaRy R. Hester L. D. Guastella D. (1999). Bilirubin, formed by activation of heme oxygenase-2, protects neurons against oxidative stress injury. Proc. Natl. Acad. Sci. U S A 96, 2445–2450. 10.1073/pnas.96.5.2445 10051662 PMC26804

[B8] Ermakov E. A. Dmitrieva E. M. Parshukova D. A. Kazantseva D. V. Vasilieva A. R. Smirnova L. P. (2021). Oxidative stress-related mechanisms in schizophrenia pathogenesis and new treatment perspectives. Oxid. Med. Cell Longev. 2021, 8881770. 10.1155/2021/8881770 33552387 PMC7847339

[B9] Fannon D. Chitnis X. Doku V. Tennakoon L. O'Ceallaigh S. Soni W. (2000). Features of structural brain abnormality detected in first-episode psychosis. Am. J. psychiatry 157, 1829–1834. 10.1176/appi.ajp.157.11.1829 11058481

[B10] Flatow J. Buckley P. Miller B. J. (2013). Meta-analysis of oxidative stress in schizophrenia. Biol. psychiatry 74, 400–409. 10.1016/j.biopsych.2013.03.018 23683390 PMC4018767

[B11] Fraguas D. Diaz-Caneja C. M. Ayora M. Hernandez-Alvarez F. Rodriguez-Quiroga A. Recio S. (2019). Oxidative stress and inflammation in first-episode psychosis: A systematic review and meta-analysis. Schizophr. Bull. 45, 742–751. 10.1093/schbul/sby125 30169868 PMC6581144

[B12] Glausier J. R. Lewis D. A. (2013). Dendritic spine pathology in schizophrenia. Neuroscience 251, 90–107. 10.1016/j.neuroscience.2012.04.044 22546337 PMC3413758

[B13] Glen A. I. Glen E. M. Horrobin D. F. Vaddadi K. S. SpellMan M. Morse-Fisher N. (1994). A red cell membrane abnormality in a subgroup of schizophrenic patients: Evidence for two diseases. Schizophr. Res. 12, 53–61. 10.1016/0920-9964(94)90084-1 8018585

[B14] Guerreiro S. Ponceau A. Toulorge D. Martin E. Alvarez-Fischer D. Hirsch E. C. (2009). Protection of midbrain dopaminergic neurons by the end-product of purine metabolism uric acid: Potentiation by low-level depolarization. J. Neurochem. 109, 1118–1128. 10.1111/j.1471-4159.2009.06040.x 19302482

[B15] Hakobyan S. Boyajyan A. Sim R. B. (2005). Classical pathway complement activity in schizophrenia. Neurosci. Lett. 374, 35–37. 10.1016/j.neulet.2004.10.024 15631892

[B16] Huang X. Lu Q. L. Zhu X. M. Zeng Y. B. Liu Y. Hu H. Y. (2021). Histogenous hypoxia and acid retention in schizophrenia: Changes in venous blood gas analysis and SOD in acute and stable schizophrenia patients. Front. Psychiatry 12, 792560. 10.3389/fpsyt.2021.792560 34938217 PMC8685331

[B17] Kaushal J. Bhutani G. Gupta R. (2012). Comparison of fasting blood sugar and serum lipid profile changes after treatment with atypical antipsychotics olanzapine and risperidone. Singap. Med. J. 53, 488–492. 22815019

[B18] Keepers G. A. Fochtmann L. J. Anzia J. M. Benjamin S. Lyness J. M. Mojtabai R. (2020), The American psychiatric association practice guideline for the treatment of patients with schizophrenia, Focus Am. Psychiatric Publ. 18, 493–497. 10.1176/appi.focus.18402 PMC7725162 33343262

[B19] Konopaske G. T. Lange N. Coyle J. T. Benes F. M. (2014). Prefrontal cortical dendritic spine pathology in schizophrenia and bipolar disorder. JAMA psychiatry 71, 1323–1331. 10.1001/jamapsychiatry.2014.1582 25271938 PMC5510541

[B20] Kutzing M. K. Firestein B. L. (2008). Altered uric acid levels and disease states. J. Pharmacol. Exp. Ther. 324, 1–7. 10.1124/jpet.107.129031 17890445

[B21] Lawrie S. M. Buechel C. Whalley H. C. Frith C. D. Friston K. J. Johnstone E. C. (2002). Reduced frontotemporal functional connectivity in schizophrenia associated with auditory hallucinations. Biol. psychiatry 51, 1008–1011. 10.1016/s0006-3223(02)01316-1 12062886

[B22] Liu H. Yu R. Gao Y. Li X. Guan X. Thomas K. (2022). Antioxidant enzymes and weight gain in drug-naive first-episode schizophrenia patients treated with risperidone for 12 Weeks: A prospective longitudinal study. Curr. Neuropharmacol. 20, 1774–1782. 10.2174/1570159X19666210920090547 34544343 PMC9881063

[B23] Lohr J. B. Browning J. A. (1995). Free radical involvement in neuropsychiatric illnesses. Psychopharmacol. Bull. 31, 159–165. 7675980

[B24] Lu Z. Wen T. Wang Y. Kan W. Xun G. (2020). Peripheral non-enzymatic antioxidants in patients with schizophrenia: A case-control study. BMC psychiatry 20, 241. 10.1186/s12888-020-02635-8 32414343 PMC7227358

[B25] Martinez B. Peplow P. V. (2020). MicroRNAs in blood and cerebrospinal fluid as diagnostic biomarkers of multiple sclerosis and to monitor disease progression. Neural Regen. Res. 15, 606–619. 10.4103/1673-5374.266905 31638082 PMC6975152

[B26] McGuinness A. J. Davis J. A. Dawson S. L. Loughman A. Collier F. O'Hely M. (2022). A systematic review of gut microbiota composition in observational studies of major depressive disorder, bipolar disorder and schizophrenia. Mol. psychiatry 27, 1920–1935. 10.1038/s41380-022-01456-3 35194166 PMC9126816

[B27] Mihai D. P. Ungurianu A. Ciotu C. I. Fischer M. J. M. Olaru O. T. Nitulescu G. M. (2021). Effects of venlafaxine, risperidone and febuxostat on cuprizone-induced demyelination, behavioral deficits and oxidative stress. Int. J. Mol. Sci. 22, 7183. 10.3390/ijms22137183 34281235 PMC8268376

[B28] Morera A. L. Henry M. García-Hernández A. Fernández-López L. GarciA-Hernandez A. (2007). Acute phase proteins as biological markers of negative psychopathology in paranoid schizophrenia. Actas espanolas Psiquiatr. 35, 249–252. 17592787

[B29] Müller N. Schiller P. Ackenheil M. (1991). Coincidence of schizophrenia and hyperbilirubinemia. Pharmacopsychiatry 24, 225–228. 10.1055/s-2007-1014472 1812499

[B30] Naureen Z. Dhuli K. Medori M. C. Caruso P. Manganotti P. Chiurazzi P. (2022). Dietary supplements in neurological diseases and brain aging. J. Prev. Med. Hyg. 63, E174–e188. 10.15167/2421-4248/jpmh2022.63.2S3.2759 PMC9710403 36479494

[B31] Noto C. Ota V. K. Gadelha A. Noto M. N. Barbosa D. S. Bonifacio K. L. (2015). Oxidative stress in drug naïve first episode psychosis and antioxidant effects of risperidone. J. Psychiatr. Res. 68, 210–216. 10.1016/j.jpsychires.2015.07.003 26228421

[B32] Owen M. J. Sawa A. Mortensen P. B. (2016). Schizophr. *Lancet* 388, 86–97. 10.1016/S0140-6736(15)01121-6 PMC4940219 26777917

[B33] Pae C. U. Paik I. H. Lee C. Lee S. J. Kim J. J. Lee C. U. (2004). Decreased plasma antioxidants in schizophrenia. Neuropsychobiology 50, 54–56. 10.1159/000077942 15179021

[B34] Patel M. (2016). Targeting oxidative stress in central nervous system disorders. Trends Pharmacol. Sci. 37, 768–778. 10.1016/j.tips.2016.06.007 27491897 PMC5333771

[B35] Pommerening Dornelles E. Gama Marques J. Ouakinin S. (2019). Unconjugated bilirubin and schizophrenia: A systematic review. CNS Spectr. 24, 577–588. 10.1017/s109285291800161x 30915934

[B36] Radhakrishnan R. Kanigere M. Menon J. Calvin S. Janish A. Srinivasan K. (2011). Association between unconjugated bilirubin and schizophrenia. Psychiatry Res. 189, 480–482. 10.1016/j.psychres.2011.03.003 21470692

[B37] Rigato I. Ostrow J. D. Tiribelli C. (2005). Bilirubin and the risk of common non-hepatic diseases. Trends Mol. Med. 11, 277–283. 10.1016/j.molmed.2005.04.008 15949769

[B38] Rossetti A. C. Paladini M. S. Riva M. A. Molteni R. (2020). Oxidation-reduction mechanisms in psychiatric disorders: A novel target for pharmacological intervention. Pharmacol. Ther. 210, 107520. 10.1016/j.pharmthera.2020.107520 32165136

[B39] Shoshina II Hovis J. K. Felisberti F. M. Santos N. A. Adreeva A. Butler P. D. (2021). Visual processing and BDNF levels in first-episode schizophrenia. Psychiatry Res. 305, 114200. 10.1016/j.psychres.2021.114200 34653830

[B40] Siciliano G. Piazza S. Carlesi C. Del CoronA A. Franzini M. PompellA A. (2007). Antioxidant capacity and protein oxidation in cerebrospinal fluid of amyotrophic lateral sclerosis. J. neurology 254, 575–580. 10.1007/s00415-006-0301-1 17426914

[B41] Smeland O. B. Frei O. Dale A. M. Andreassen O. A. (2020). The polygenic architecture of schizophrenia - rethinking pathogenesis and nosology. Nat. Rev. Neurol. 16, 366–379. 10.1038/s41582-020-0364-0 32528109

[B42] Solberg D. K. Refsum H. Andreassen O. A. Bentsen H. (2019). A five-year follow-up study of antioxidants, oxidative stress and polyunsaturated fatty acids in schizophrenia. Acta neuropsychiatr. 31, 202–212. 10.1017/neu.2019.14 31178002

[B6] Spencer L J. Degu A. Kalkidan H. A. Solomon M. A. Cristiana A. Nooshin A. (2018). Lancet. 392, 1789–1858. 10.1016/s0140-6736(18)32279-7 30496104 PMC6227754

[B43] Valli I. Crossley N. A. Day F. Stone J. Tognin S. Mondelli V. (2016). HPA-axis function and grey matter volume reductions: Imaging the diathesis-stress model in individuals at ultra-high risk of psychosis. Transl. Psychiatry 6, e797. 10.1038/tp.2016.68 27138796 PMC5070043

[B44] Vetulani J. (2013). Early maternal separation: A rodent model of depression and a prevailing human condition. Pharmacol. Rep. 65, 1451–1461. 10.1016/s1734-1140(13)71505-6 24552992

[B45] Wayner D. D. Burton G. W. Ingold K. U. Barclay L. R. Locke S. J. (1987). The relative contributions of vitamin E, urate, ascorbate and proteins to the total peroxyl radical-trapping antioxidant activity of human blood plasma. Biochimica biophysica acta 924, 408–419. 10.1016/0304-4165(87)90155-3 3593759

[B46] Wu X. Niu Z. Zhu Y. Shi Y. Qiu H. Gu W. (2022). Peripheral biomarkers to predict the diagnosis of bipolar disorder from major depressive disorder in adolescents. Eur. archives psychiatry Clin. Neurosci. 272, 817–826. 10.1007/s00406-021-01321-4 34432143

[B47] Yao J. K. Keshavan M. S. (2011). Antioxidants, redox signaling, and pathophysiology in schizophrenia: An integrative view. Antioxid. Redox Signal 15, 2011–2035. 10.1089/ars.2010.3603 21126177 PMC3159108

[B48] Yao J. K. Reddy R. van Kammen D. P. (2000). Abnormal age-related changes of plasma antioxidant proteins in schizophrenia. Psychiatry Res. 97, 137–151. 10.1016/s0165-1781(00)00230-4 11166086

